# Parental Concerns About their Child’s Development During the First Year of Life and a Subsequent Autism Spectrum Disorder Diagnosis

**DOI:** 10.1007/s10803-025-06923-8

**Published:** 2025-06-18

**Authors:** Orly Kerub, Einav Alhozyel, Rewa Blaaum, Leena Elbedour, Gal Meiri, Dikla Zigdon, Analya Michaelovski, Ronnie Frankel, Mira Sopitsky Goshen, Michael Gdalevich, Nadav Davidovich, Idan Menashe

**Affiliations:** 1 Southern District Health Office, Ministry of Health, Beer-Sheva, Israel; 2 Department of Health Systems Management, Faculty of Health Sciences, Ben-Gurion University of the Negev, Beer-Sheva, Israel; 3 Department of Epidemiology, Biostatistics and Community Health Sciences, Faculty of Health Sciences, Ben-Gurion University of the Negev, 8410501 Beer-Sheva, Israel; 4 Azrieli National Center for Autism and Neurodevelopment Research, Ben-Gurion University of the Negev, Beer-Sheva, Israel; 5 Preschool Psychiatric Unit, Soroka University Medical Center, Beer Sheva, Israel; 6 Child Development Center, Soroka University Medical Center, Beer-Sheva, Israel

**Keywords:** Autism spectrum disorder, Parental concerns, Child development, Developmental milestones

## Abstract

Eliciting parents’ concerns about their child’s development is a key component of developmental surveillance. Here, we aimed to examine whether parental concern about the child’s development during the first year of life is associated with a later diagnosis of autism spectrum disorder (ASD). We compared prospectively collected data from medical records on parental concerns and children’s developmental milestones at ages 2, 4, 6, 9, and 12 months between 280 children later diagnosed with ASD and 560 children without ASD. Overall, 8.1% of parents in our study expressed concerns regarding their child’s development during the first year of life, with a significantly higher portion of parents of children later diagnosed with ASD expressing concerns vs. parents of children without ASD (19.5% vs. 2.8%; p < 0.001). Notably, parental concerns were associated with the failure of children to master age-appropriate language, motor, and social developmental milestones (OR = 5.27, p < 0.001; OR = 2.46, p = 0.023; and OR = 2.27, p = 0.012, respectively). Nevertheless, even after adjustment for this association, parental concerns were found to be an independent risk factor for ASD (aOR = 7.76; 95%CI = 4.31–13.97). Thus, regular monitoring of parent-reported concerns may be invaluable in early screening programs for ASD.

**Supplementary Information:**

The online version contains supplementary material available at https://doi.org/10.1007/s10803-025-06923-8.

Currently, the diagnosis of autism spectrum disorder (ASD) relies on the clinical evaluation of impairments in social reciprocity and communication and the manifestation of stereotypical patterns of behavior and interests, which are usually not apparent until the second year of life (Baio et al., 2018). Consequently, most children with ASD are diagnosed only after 3 years of age (Shaw et al., 2020). Nonetheless, early diagnosis of ASD has immense implications for prognosis: It has been convincingly demonstrated that the effect of most interventions designed to ameliorate ASD symptoms is strongly correlated with the age of the child at the time interventions are instituted, with earlier interventions leading to better outcomes (Bradshaw et al., 2015; Gabbay-Dizdar et al., 2022; Hyman et al., 2020; Rogers et al., 2014; Sandbank et al., 2020; Towle et al., 2020; Whitehouse et al., 2019; Zwaigenbaum et al., 2015). This evidence thus calls for better screening approaches that will subsequently lead to earlier ASD diagnosis (Bradshaw et al., 2015; Dawson, 2008; Sullivan et al., 2014; Zwaigenbaum et al., 2015).

Eliciting parents’ concerns about their child’s development is a key component of developmental surveillance (Dosreis et al., 2006; Volkmar et al., 2014). In previous studies, about 50% of parents of a child with ASD reported that they had had concerns before 12 months of age, due to observed abnormalities in social interactions and also in other developmental domains, including motor skills, attention, and temperament (Clifford & Dissanayake, 2008; De Giacomo & Fombonne, 1998; Gillberg et al., 1990; Ozonoff et al., 2009; Watson et al., 2007). However, most studies of parental concern in this context were based on retrospective questionnaires about the child’s development (Becerra-Culqui et al., 2018; Zablotsky et al., 2017), while only a few studies used data that was collected prospectively from parents during the first 12 months of their children’s lives (Ozonoff et al., 2009; Sacrey et al., 2015, 2018; Tran et al., 2021). Furthermore, those prospective studies were conducted on high-risk families (i.e., families with a child already diagnosed with ASD), with such parents usually being particularly alert to their child’s developmental trajectories. Thus, further research on this matter using prospective data collected from a population-representative sample is warranted.

To address the above knowledge gap, we examined the association between parental concerns about their child’s development during the first year of life and a later diagnosis of ASD by leveraging large-scale prospective data collected in the framework of regular child developmental monitoring at mother–child health clinics (MCHCs) in the Negev area (southern Israel).

## Methods

### Setting

The study was conducted in 47 government-funded MCHCs that provide health services to > 95% of children aged 0–6 years in southern Israel. This area is defined by a population of approximately one million residents of two main ethnicities Jews and Bedouin Arabs. In these MCHCs, trained nurses provide health promotion and disease prevention services for pregnant women, infants and children (Israel Ministry of Health, 2023). In addition, they routinely monitor the development of children born in the area via the evaluation of age-appropriate developmental milestones. In the beginning of each evaluation sessions, the nurses ask the parents if they have any concerns about their child’s development and record the answers in the child’s medical charts. Nurses in MCHCs located in Bedouin villages, speaks both Hebrew and Arabic and hence communicate with the children and their parents in their preferred language. Importantly, children may skip one or more scheduled visits at MCHC clinics due to medical or other reasons.

### Study Sample

The study sample was ascertained from all children who were born between January 2014 and July 2018 and whose development was monitored in one of the 47 MCHCs in the Negev. We included only children who are members of Clalit Health Services (CHS), the largest health service provider in Israel, serving ~ 75% of the Negev population. Importantly, child members of CHS who fail to master one or more developmental milestones at their MCHC visits are referred for further evaluation at Soroka University Medical Center (SUMC). There, they undergo comprehensive developmental assessments until a diagnosis is made (may include ASD, or other developmental delays) (Kerub et al., 2020; Meiri et al., 2017). For this study, we randomly selected 280 out of the ~ 500 children with ASD who were diagnosed at SUMC and used their national identity numbers to retrieve their developmental records from the MCHCs. In addition, data for children without ASD or any other developmental disorder was ascertained from the same MCHCs to be used in this study as the comparison group—designated the no-ASD comparison group. The no-ASD group was matched to the ASD group in a 2:1 ratio to increase statistical power as no-ASD children are remarkably more prevalent than ASD children in the population. The matching of the no-ASD to ASD children was on the basis of date of birth (± 3 months), sex (male/female) and ethnicity (Jewish/Bedouin). We did not include children who had been lost to follow-up (missing ≥ 2 checkups) and excluded two children who had been referred to SUMC for further developmental evaluation but had not completed the developmental assessment at the time of data collection. This sample size has been shown to have sufficient statistical power (80%) to detect differences of two-folds or more in parental concerns between cases and controls.

### Data Collection

Data collection was completed in December 2022. Data regarding the children’s birth and achievements at age-appropriate developmental milestones and the parents’ socioeconomic status and concerns regarding the development of their children was retrieved manually from the MCHCs’ electronic charts. Specifically, data regarding the children’s development and the concerns of parents recorded at the first five checkup appointments at the MCHC clinics was collected. At each meeting, the MCHC nurse would ask the parents: “Do you have any concerns about your child’s development?” In this study, we investigated whether the parents’ answer to this question, namely, parental concerns, could be used as an indicator of a later diagnosis of ASD.

### Statistical Analysis

We used chi-square and T-tests to assess statistically significant differences in the collected variables between the study groups. In addition, odds ratios with 95% confidence intervals (CI) were used to assess the odds of parental concerns about the child’s development being associated with a later diagnosis of ASD. Finally, logistic regression models were used to assess the independent association of parental concerns at specific ages with the risk of ASD, while adjusting for ≥ 1 failed milestones. Statistical significance was set at a threshold of α = 0.05.

### Community Involvement Statement

The research team included leading autism practitioners and policymakers. There was no other community involvement in this work.

## Results

The study sample included 840 children (280 with ASD and 560 without ASD); 78.2% of the children were males and 23.2% were of Bedouin origin. The basic sociodemographic and clinical characteristics of study participants are given in Table 1. Parents of ASD children were, on average, one year older than parents of no-ASD children (30.58 ± 5.99 vs. 29.68 ± 5.53; p = 0.031 and 33.86 ± 6.91 vs. 32.69 ± 6.53; p = 0.020 for mothers and fathers of ASD and no-ASD children, respectively). In addition, a substantially lower portion of the ASD children were families with higher socioeconomic status compared to their no-ASD counterparts (3.6% vs. 22.3%; p < 0.001). Finally, ASD vs. no-ASD children were more likely to be born by Cesarean Sect. (28.7% vs. 18.4%; p = 0.002), at an earlier gestational age (38.50 ± 2.91 vs. 39.54 ± 1.33 weeks; p < 0.001), and at a lower gestational weight (3.06 ± 0.676 vs. 3.31 ± 0.388; p < 0.001).

**Table 1:** Characteristics of the study sample

Variable	ASD (n = 280)	Non-ASD (n = 560)	p value
Gender (male), N (%)	219 (78.2%)	438 (78.2%)	1^a^
Ethnicity (Jewish), N (%)	215 (76.8%)	430 (76.8%)	1^a^
Socio-economic status, N (%)			
Low	95 (33.9%)	180 (32.1%)	< 0.001^a^
Moderate	175 (62.5%)	255 (45.5%)	
High	10 (3.6%)	125 (22.3%)	
Mother’s age at birth (years), mean ± SD	30.58 ± 5.99	29.68 ± 5.53	0.031^b^
Father’s age at birth (years), mean ± SD	33.86 ± 6.91	32.69 ± 6.53	0.020^b^
Birth details, N (%)			
Spontaneous	205 (73.2%)	442 (78.9%)	Ref
Cesarean section	68 (24.3%)	86 (15.4%)	0.002^a^
Assisted delivery	7 (2.5%)	32 (5.7%)	0.093^a^
Birth week, mean ± SD	38.50 ± 2.91	39.54 ± 1.33	< 0.001^b^
Premature delivery (< 37 weeks), N (%)	46 (17.2%)	26 (4.7%)	< 0.001^a^
Birth weight (g), mean ± SD	3.06 ± 0.676)	3.31 ± 0.388	< 0.001^b^

^a^Chi-square test

^b^Two-sided unpaired T-test

### Parental Concerns

**Fig. 1:**
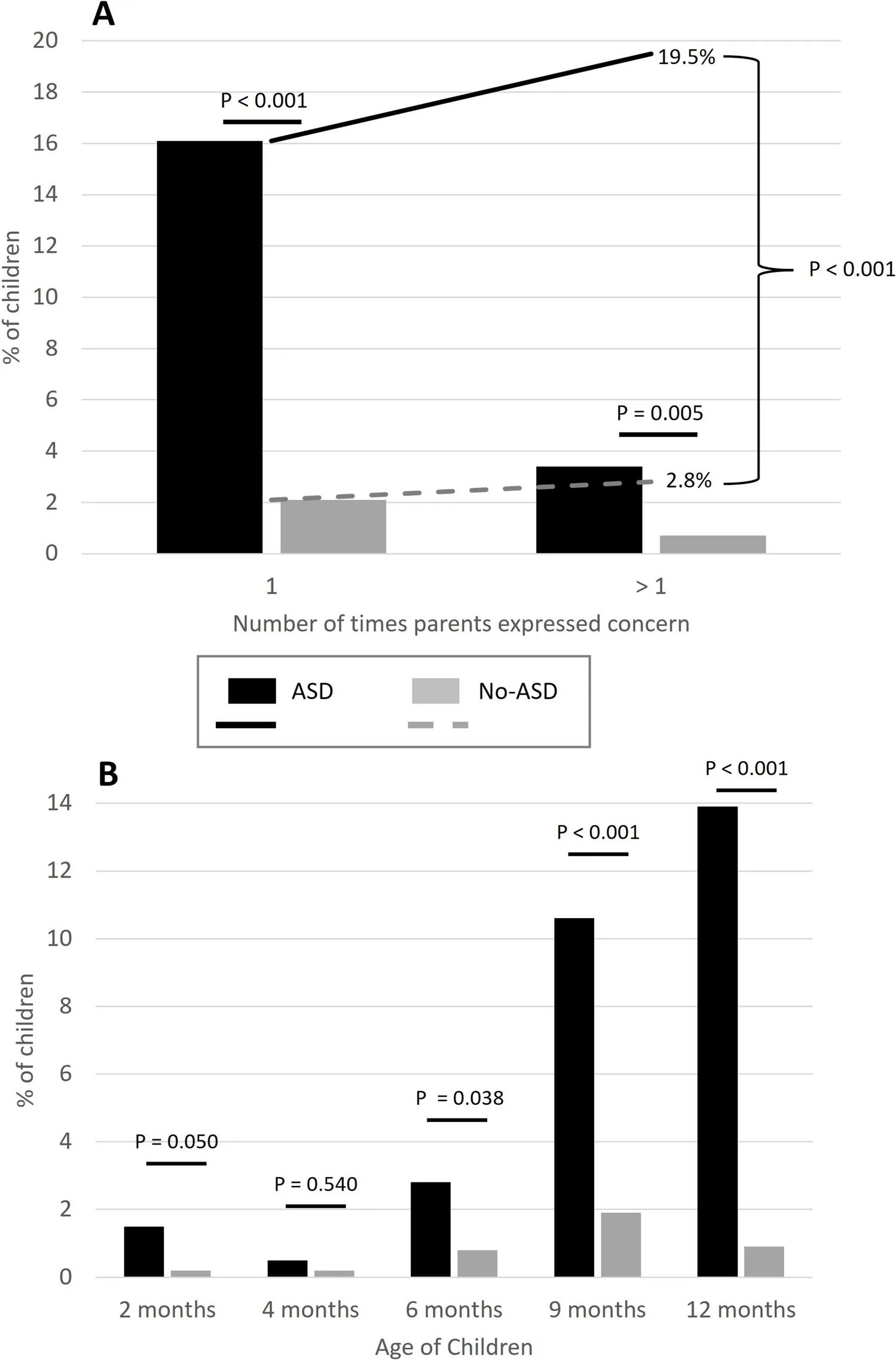
Differences in the proportions of children whose parents expressed concerns regarding their development between ASD cases (black bars/ solid lines) and no-ASD controls (gray bars/dashed lines). **A** A total of 19.5% of parents of children later diagnosed with ASD raised concern regarding their development (16% raised concerns only once and another 3.5% more than once) vs. only 2.8% of parents of no-ASD children (2%, once and 0.8% more than once respectively). **B** Differences in the proportions of children whose parents expressed concerns regarding their development between cases and controls are presented for each age of which developmental assessment was conducted during their first year of life. P values indicate the statistical significance of the differences between cases and controls

Parents of 68 children (8.1%) expressed concerns about their children’s development during the first year of life. Of these, the parents of 55 children expressed concerns only once, while the parents of 13 expressed concerns two or more times during that time. There were significant differences between the study groups with regard to the number of parents who had raised concerns about their child’s development: parents of 19.5% of children later diagnosed with ASD had raised concerns, compared to only 2.8% of parents of non-ASD children (p < 0.001; Fig. 1A). There were also group differences in the number of times concerns were raised by parents, with 3.4% of parents of ASD children raising concerns multiple times compared to only 0.7% of parents of no-ASD children (p = 0.005; Fig. 1A).

An examination of parental concerns expressed in the five visits to the MCHCs during the babies’ first year of life (at the ages of 2, 4, 6, 9, and 12 months) revealed no significant group differences in the ages at which children visited the MCHCs (Supplementary Table S1). Figure 1B shows the proportions of children whose parents expressed concerns during these visits. As expected, more parents expressed concerns about their children’s development as the children became older. Here, too, higher rates of parents of children later diagnosed with ASD expressed concerns about their child’s development at all ages than their counterparts, with larger differences being seen at older ages (Fig. 1B).

### Association Between Parental Concerns and Developmental Milestone Failures

**Fig. 2:**
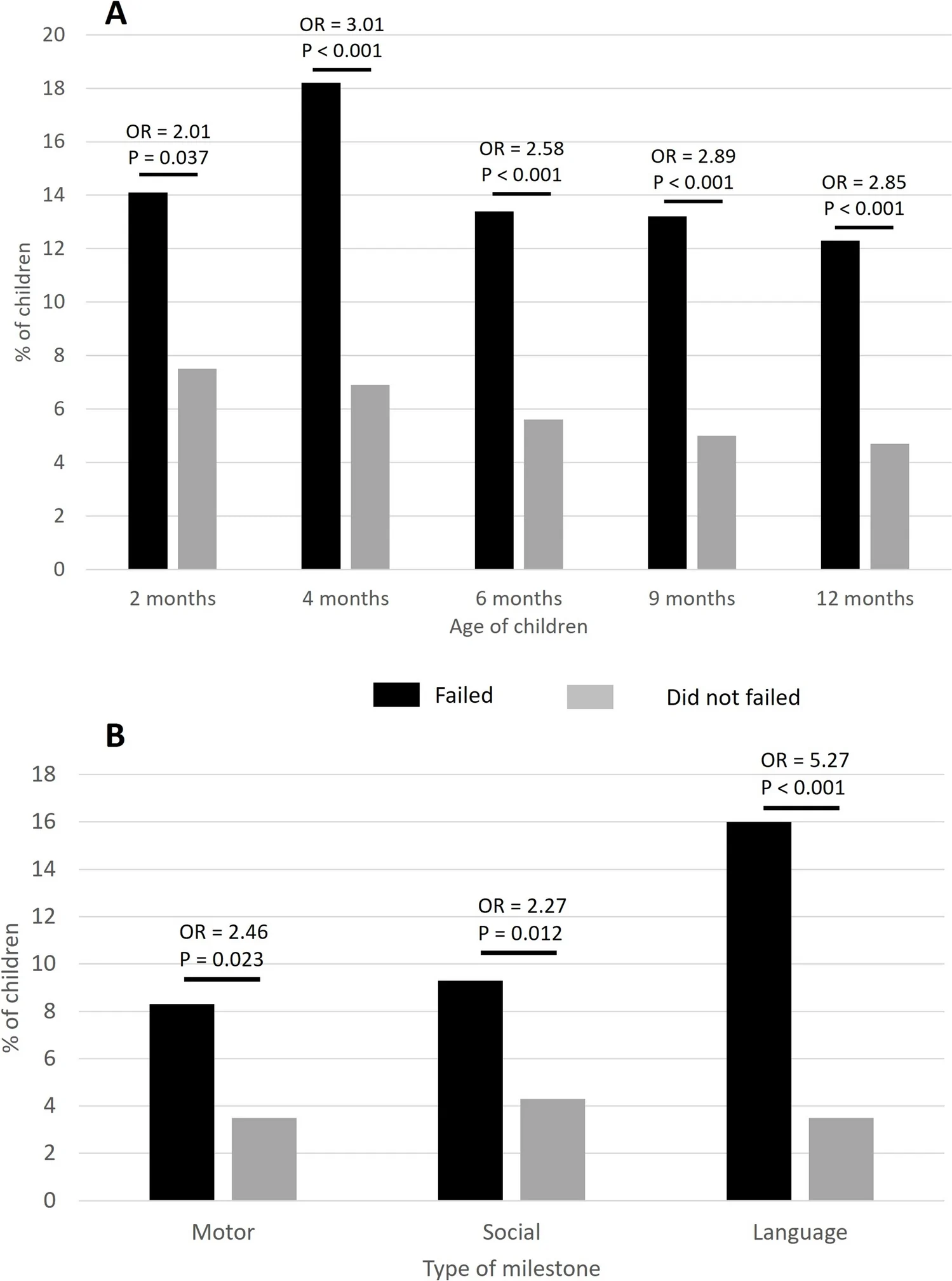
Association between parental concerns and failure in developmental milestones during the first year of life. The percentage of children whose parents expressed concern regarding the development of their child during the first year of life are compared between children who failed (black bars) and did not fail (gray bars) in at least one age-appropriate developmental milestone. Odds ratios (ORs) and corresponding p values for the association between parental concern and developmental milestone failure are provided for different ages at which developmental milestones were examined (**A**) and for the different developmental milestone categories (**B**)

We sought to reveal whether parental concerns regarding their child’s development was associated with failure in mastering developmental milestones, as assessed at the MCHCs. Overall, at each of the MCHC visits during the first year of life, parents of children who failed to master at least one developmental milestone were 2–3 times more likely to express concerns about their child’s development than parents of children who did not fail (Fig. 2A). Interestingly, parental concerns were mostly associated with failure in language acquisition milestones (OR = 5.27; p < 0.001; Fig. 2B), especially towards the end of the first year of life (supplementary Table S2), implying that language was the most significant factor in the child’s development that elicited the concern of parents of children later diagnosed with ASD.

### Association Between Parental Concerns Regarding Developmental Milestone Failure and ASD

Finally, we tested the association between parental concerns regarding developmental milestone failure and a later diagnosis of ASD, with and without adjustment for the child’s failure in one or more developmental milestones, social-economic status, maternal and paternal ages, cesarean section, and birth week and weight (Table 2). Overall, children of parents who expressed concerns during the first year of life had ~ 7 times higher odds to be diagnosed with ASD compared to children of parents who did not express concerns, even after adjustment for developmental milestone failure (aOR = 7.12; 95%CI = 3.57–14.18). These differences were seen among both Jewish and Bedouin parents (Supplementary Table S3) This association was most marked towards the end of the first year of life at 9 and 12 months of age (aOR = 4.57; 95%CI = 1.76–11.84, and aOR = 11.72; 95%CI = 3.89–35.35 respectively).

**Table 2:** Association between parental concerns during the first year of life and a subsequent ASD diagnosis

Age (months)	N	OR	95% CI of OR	Adjusted OR	95% CI of OR
Any	840	8.22	4.59–14.72	7.12	3.57–14.18
2	643	7.04	0.73–68.08	4.53	0.36–56.80
4	667	2.33	0.15–37.36	0.44	0.01–15.44
6	711	3.56	0.99–12.73	1.95	0.42–9.02
9	699	6.15	2.78–13.60	4.57	1.76–11.84
12	802	17.79	6.87–46.09	11.72	3.89–35.35

Logistic regression adjusted to failed ≥ 1 milestones, Social-economic status, Maternal age, Paternal age, Cesarean section, Birth week and Birth weight

## Discussion

This study aimed to examine whether parental concerns about their child’s development during the first year of life are associated with ASD risk. To the best of our knowledge, this is the first study to use prospectively documented data from medical charts of a population-based sample for this purpose. While only 8% of parents expressed concerns regarding the development of their children during their first year of life, our findings show that such parental concerns may serve as an independent indicator of a later diagnosis of ASD. Children of parents who expressed concerns were ~ 8 times more likely to be subsequently diagnosed with ASD than children of parents who had not expressed concerns during the same period. These findings are in line with the findings of several studies that used retrospective questionnaires at the time of ASD diagnosis, with the retrospective studies also finding that parents of children diagnosed with ASD were more likely to be concerned about their child’s development than parents of children without ASD (Cleary et al., 2024; Richards et al., 2016; Zuckerman et al., 2015). Similarly, several prospective studies that also assessed the relationship between parental concerns and a later diagnosis of ASD reported that parents in high-risk families, i.e., families with one or more children already diagnosed with ASD, were more likely to be concerned about the development of the other children in the family than parents with no ASD children in the family (Hess & Landa, 2012; Ozonoff et al., 2009; Sacrey et al., 2015). This indicates that parents who already have a child with ASD are more likely to be hypervigilant for early developmental abnormalities predisposing to ASD development (Garrido et al., 2018; Sacrey et al., 2015). Unfortunately, in our analyses, information regarding an ASD diagnosis for other children in the family was not available to us and thus adjustment for this important factor was not possible.

Our findings suggest that there is indeed an association between the failure of children to master developmental milestones during the first year of life, particularly those related to language acquisition and motor and social development, and parental concerns. These findings reiterate previous research pinpointing parental concerns regarding failure in language milestones as a plausible predictor of an ASD diagnosis (De Giacomo & Fombonne, 1998; Herlihy et al., 2015; Hess & Landa, 2012; Matheis et al., 2017; Richards et al., 2016; Turygin et al., 2014). Of note, delays in sensory and motor development have also been reported as leading to concerns of parents of children later diagnosed with ASD (Sacrey et al., 2015). Taken together, these findings may suggest that parents’ awareness regarding the timing of milestone acquisition in various developmental categories may elicit concerns about their child’s development regardless of the potential manifestation of early autism symptoms. Nonetheless, despite the association between parental concern and failure in developmental milestones, parental concern was shown to be an additional risk factor for ASD diagnosis in the present study, thereby validating parent-reported concerns as an essential tool in the early identification of ASD. 

The major strength of this study lies in the use of prospectively documented data from medical charts of a population-based sample, which facilitates the generalizability of the findings and reduces the risk of recall bias. Nevertheless, our study has several limitations that should also be taken into consideration. First, because children may skip one or more scheduled visits at MCHC clinics due to medical or other reasons, we had slightly different sample sizes for different ages in the study. Nevertheless, because our sample did not include children who skipped more than two appointments and because skipping an appointment is likely to be a random event, such missing data should not have a significant effect on our results. Second, a limited number of parents expressed concerns regarding their child’s development, generating a relatively small sample size. In addition, our sample was drawn from only one region of the country, which may not be a reliable representation of the country’s overall population. Thus, our findings should be confirmed in a larger and more population-representative sample. Third, as our dataset was based on data collected from the medical charts of children in MCHC clinics, it did not include information regarding siblings diagnosed with ASD. It is, therefore, conceivable that this sample included parents of older children diagnosed with ASD, possibly contributing to the higher proportion of parental concerns observed in the ASD group. 

## Conclusions

These findings suggest that parental concerns regarding their child’s development during the first year of life may be a risk factor for a later diagnosis of ASD. Therefore, regular monitoring of parent-reported concerns may be invaluable in early screening programs for ASD.

## Supplementary Information

Below is the link to the electronic supplementary material.

Supplementary file1 (DOCX 18 KB)
