# Supplementary material for: Parental Concerns About their Child’s Development During the First Year of Life and a Subsequent Autism Spectrum Disorder Diagnosis
Source: J Autism Dev Disord. 2025 Jun 18;56(12):4874–81. doi: 10.1007/s10803-025-06923-8 (PMC13694329; doi:10.1007/s10803-025-06923-8)
Supplement: Supplementary file 1 [file 10803_2025_6923_MOESM1_ESM.docx]

**Table S2:** Association between parental concerns and failure in developmental milestones across different ages during the first year of life.

| **Failures in language milestones** | | | | **Failures in social milestones** | | | | **Failures in motor milestones** | | | | **Age** |
| --- | --- | --- | --- | --- | --- | --- | --- | --- | --- | --- | --- | --- |
| **p-value** | **OR** | **no** | **yes** | **p-value** | **OR** | **no** | **yes** | **p-value** | **OR** | **no** | **yes** |  |
| <0.001 | 5.27 | 18  3.5% | 26  16.0% | 0.012 | 2.27 | 15  4.3% | 27  9.3% | 0.023 | 2.46 | 8  3.5% | 29  8.3% | **Any** |
| 0.819 | 0.99 | 4  0.6% | 0  0.0% | <0.001 | 113.25 | 1  0.2% | 3  15.8% | <0.001 | 23.91 | 1  0.2% | 3  4.2% | **2 months** |
| 0.861 | 0.99 | 2  0.3% | 0  0.0% | 0.855 | 0.97 | 1  0.2% | 0  0.0% | <0.001 | 0.12 | 2  2.4% | 0  0.0% | **4 months** |
| 0.198 | 3.67 | 8  1.2% | 1  4.2% | - | - | 0 | 0 | 0.032 | 4.90 | 2  0.5% | 6  2.3% | **6 months** |
| <0.001 | 6.68 | 15  2.5% | 12  14.5% | <0.001 | 8.49 | 14  2.3% | 14  16.9% | <0.001 | 4.69 | 7  1.7% | 21  7.5% | **9 months** |
| <0.001 | 9.90 | 14  2.1% | 24  17.6% | <0.001 | 12.57 | 4  0.9% | 33  10.0% | <0.001 | 11.72 | 17  2.4% | 22  22.7% | **12 months** |
